# Evaluation of the Circadian Rhythm Component Cipc (Clock-Interacting Pacemaker) in Leukemogenesis: A Literature Review and Bioinformatics Approach

**DOI:** 10.3390/clockssleep7030033

**Published:** 2025-06-25

**Authors:** Leidivan Sousa da Cunha, Beatriz Maria Dias Nogueira, Flávia Melo Cunha de Pinho Pessoa, Caio Bezerra Machado, Deivide de Sousa Oliveira, Manoel Odorico de Moraes Filho, Maria Elisabete Amaral de Moraes, André Salim Khayat, Caroline Aquino Moreira-Nunes

**Affiliations:** 1 Department of Medicine, Clinical Genetics Laboratory, Drug Research and Development Center (NPDM), Federal University of Ceará, Fortaleza 60430-275, CE, Brazil; leidivansc@gmail.com (L.S.d.C.); bmdnogueira@gmail.com (B.M.D.N.); flaviamelop@outlook.com (F.M.C.d.P.P.); caio.bmachado97@gmail.com (C.B.M.); deividearmorial@gmail.com (D.d.S.O.); odorico@ufc.br (M.O.d.M.F.); betemora@ufc.br (M.E.A.d.M.); 2 Department of Biological Sciences, Oncology Research Center, Federal University of Pará, Belém 66077-830, PA, Brazil; khayatas@gmail.com; 3 Brazilian Institute of Intelligence in Health, Research and Education, IBISPE, Fortaleza 60160-230, CE, Brazil; 4 Clementino Fraga Group, Central Unity, Molecular Biology Laboratory, Fortaleza 60455-970, CE, Brazil

**Keywords:** circadian rhythms, leukemia, gene expression

## Abstract

Circadian rhythms (CRs) are a key biological system regulating physiological processes such as metabolism, cell growth, DNA repair, and immunity, adapting to environmental changes like the light/dark cycle. Governed by internal clocks, it modulates gene expression through feedback loops involving Clock Genes (CGs), with the cycle initiated by CLOCK–BMAL1 and NPAS2–BMAL1 heterodimers. Disruptions in circadian rhythms have been linked to diseases including metabolic disorders, neurodegeneration, and cancer. CIPC (CLOCK-interacting pacemaker) has been studied as a negative regulator of the CLOCK–BMAL1 complex, focusing on its role in cancer, particularly leukemias. Public datasets and bioinformatics tools were used to examine *CIPC* gene expression in healthy patients and acute myeloid leukemia (AML) samples. Our analysis revealed significant overexpression of *CIPC* in AML compared to healthy tissues (*p* < 0.0001 ****). Additionally, survival analysis indicated significant differences in overall survival based on *CIPC* expression, with a log-rank test *p*-value = 0.014, suggesting that *CIPC* expression may affect overall patient survival. Altered *CIPC* expression may contribute to leukemogenesis by inhibiting circadian genes, which are often disrupted in leukemia. Furthermore, *CIPC* interacts with oncogenic pathways, including the MAPK/ERK pathway, which is essential for cell proliferation. Additional studies are needed to validate these findings and explore the detailed role of *CIPC* in cancer development.

## 1. Introduction

Circadian rhythms (CRs) are a cyclical system that regulate several physiological processes, maintaining homeostasis in response to external changes such as the light/dark cycle caused by the Earth’s rotation. These external stimuli are known as Zeitgebers (ZTs) [1,2,3]. These rhythms are regulated by internal clocks, which also play a role in organizing internal processes, influencing the expression of multiple genes involved in metabolism and proliferation, DNA repair, responses to environmental signals, and immunity [4,5,6].

Studies from the 1980s were pioneering in describing one of its components, known as clock genes (CGs): the *PERIOD* gene (*PER*) in the fruit fly Drosophila melanogaster, and later *CLOCK* in mice [7,8,9,10].

In this sense, CRs are divided into two operational levels; this includes a systemic one, located in the suprachiasmatic nucleus (SCN) of the central nervous system, which responds to ZTs at sunrise and stimulates the transcription of CG in peripheral tissues [6,11,12,13].

This expression of CGs in peripheral tissues is referred to as the cellular level, which is positively driven by the transcription of CGs, such as *CLOCK*, *BMAL1*/*ARNTL*, and NPAS2. These genes produce heterodimers known as CLOCK–BMAL1 and NPAS2–BMAL1. These proteins bind to enhancer boxes (E-boxes) in their target promoters and stimulate the expression of other CGs, such as *PER1/2/3*, *CRY1/2*, and *TIM* [14,15,16,17].

Throughout the day, the levels of clock proteins continue to increase. When highly expressed, they associate with casein kinase 1 (CK1ε) and are then transported into the cell nucleus, where they bind to heterodimers, such as CLOCK–BMAL1 and NPAS2–BMAL1. This leads to inhibition and negative regulation of the cycle by the end of the day, a process known as a positive and negative transcription–translation feedback loop (TTFL) [12,14,16,18,19,20].

## 2. Clock Genes and Disease

These physiological and molecular functions of CRs are extremely important for maintaining homeostasis by efficiently orchestrating metabolic processes such as anabolism and catabolism. Therefore, the association between circadian dysregulation and metabolic disorders is well established [21].

In this context, shift work and CG variants have been associated with an increased risk of type 2 diabetes [22]. Additionally, other circadian disruptions influence hyperinsulinemia and insulin resistance, further contributing to the onset of the disease [23]. In addition to metabolic changes, dysregulation of the biological clock also contributes to cognitive impairment and neurodegeneration. This has been observed in studies of sleep-deprived mice with Alzheimer’s disease, which showed altered levels of BMAL1 protein, as well as in patients with Parkinson’s disease, in whom relative BMAL1 levels positively correlate with disease severity [24,25].

In addition to clear interactions with metabolic events, several studies have shown circadian regulation of the cell cycle (CC) [26]. In this context, clock genes (CGs) act as critical regulators at different phases, where they can either stimulate or inhibit cell proliferation [26,27]. Furthermore, the cell cycle itself includes genes that are regulated by CGs, known as clock-controlled genes (CCGs), such as *MYC*, *P21*, and cyclins [28,29,30,31].

### 2.1. Clock Genes and Cancer

Dysregulation of the CC and metabolic disorders are well-established hallmarks of neoplastic development and maintenance [32,33,34,35]. Accordingly, researchers have begun to investigate the links between circadian disruption and oncogenesis, such as the tumor-suppressor roles of *BMAL1* and *PER2* in lung tumor initiation and progression, and the induction of MYC by CLOCK and bHLH proteins, which facilitates neuroblastoma progression [36,37].

Other studies have also associated shift work and alterations in CGs with an increased risk of developing skin tumors, including squamous cell carcinoma, melanoma, and basal cell carcinoma, as well as gastric cancer [38,39]. Furthermore, genomic integrity may be compromised due to the influence of *CLOCK*, *BMAL1*, *PER*, and *CRY* on key signaling pathways such as c-Myc/p21 and Wnt/β-catenin, potentially leading to impaired DNA damage responses in cancer [40,41]. Additional findings suggest that the master regulators CLOCK and BMAL1 possess anti-apoptotic functions that contribute to the proliferation of liver cancer cells, while their inhibition leads to dysregulation of *WEE1* and *p21*, ultimately promoting tumor cell death [42].

### 2.2. Clock Genes and Oncohematologic Neoplasms

Leukemias are a group of hematological malignancies characterized by the clonal expansion of malignant cells in the bone marrow, originating from either the myeloid or lymphoid lineage [43].

Leukemic cells can appear in several forms: they may be predominantly mature, predominantly immature, or a mixture of both maturation stages. Leukemia is generally categorized into four main types: acute myeloid leukemia (AML) and acute lymphoblastic leukemia (ALL), which are characterized by the presence of immature cells; chronic lymphocytic leukemia (CLL), which involves predominantly mature cells; and chronic myeloid leukemia (CML), which may present a mixture of mature and immature cells, depending on the disease stage and progression [44,45].

The exact cause of leukemia remains poorly understood as it is a multifactorial disease arising from the interaction between genetic and environmental factors. In most cases, leukemia develops as a de novo malignancy in previously healthy individuals and originates from the oncogenic transformation of a hematopoietic stem cell or one of its progenitor cells [44,46].

Leukemia can be associated with mutations or genetic alterations in signaling molecules and transcription factors, leading to enhanced signal transduction and increased cell proliferation. Consequently, the critical role of these altered pathways and other genetic factors in essential cellular processes makes them valuable biomarkers and therapeutic targets for clinical research and leukemia management [47].

The CR plays a critical daily role in maintaining hematopoietic cell production in the bone marrow. Disruption or alteration of this cycle may not only be associated with the onset of leukemia but also influence its development, including leukemogenesis [48,49].

Low expression of *CLOCK* and *BMAL1*, two key genes associated with the circadian clock, has been observed in leukemias. This suggests that deregulated circadian mechanisms may facilitate the proliferation and maintenance of leukemic cells, highlighting the need to evaluate CG regulatory pathways. Particularly concerning is the consistent negative regulation of core clock genes, which appears across all types of leukemia [50].

Another important finding is regulation of the CR in stem cells in acute myeloid leukemia (AML), and how its disruption can have beneficial effects, such as impaired proliferation and depletion of leukemic stem cells [51]. It is worth noting that circadian dysregulation, assessed by altered CG expression, has been observed in all other types of leukemia. These findings support assumptions about its possible roles in leukemogenesis, including increased aggressiveness, poorer prognosis, regulation of the cell cycle, and response to DNA damage. Circadian genes may also function as tumor suppressors, thereby contributing to the pathophysiology of leukemia [50].

## 3. CLOCK-Interacting Pacemaker (CipC)

CLOCK-interacting pacemaker (CIPC) is a key regulator in the mammalian circadian clock system. This protein functions as a negative feedback regulator that specifically inhibits the activity of the CLOCK–BMAL1 complex, a critical component of the circadian machinery. Notably, CIPC exerts its inhibitory effect independently of other negative regulators in the circadian system, such as CRYs [52,53,54].

The functional relevance of CIPC’s interaction with other signaling pathways remains unclear, but it may modulate the turnover of phosphorylated proteins through its effect on the Erk pathway, thereby influencing the activity and localization of Erk targets. Although a direct role for *CIPC* in human disease has not been established, its influence on cell proliferation and Erk signaling suggests potential relevance to disorders involving abnormal cell growth, such as cancer. These findings support additional physiological roles for *CIPC* beyond circadian regulation as demonstrated in cell-based and knockout models [52].

This work reviews the relatively little-known negative regulator of the circadian cycle, CIPC, emphasizing its significant role in cancer, particularly leukemia. Although its precise impact remains uncertain at this time, this complexity is likely crucial for the precision and robustness of the mammalian circadian system [54,55,56].

## 4. Methodology

Expression gene data were downloaded from GTEx (Genotype-Tissue Expression) for normal tissues and from the Xena Browser for neoplastic tissues, specifically expression data in AML (Project data TCGA-LAML). Statistical analyses were performed on these data, first assessing normality using the Kolmogorov–Smirnov test, followed by the non-parametric Mann–Whitney test. All statistical analyses were conducted using GraphPad Prism version 8.0.1, with significance levels set at *p* < 0.05 (95%) and *p* < 0.001 (99%).

Kaplan–Meier survival analysis was conducted using data retrieved from the Xena Browser and analyzed with Jamovi software version 2.3.28, employing its parameters to assess survival differences based on CIPC expression levels in AML. Interpretation of overall survival (OS) data was performed using the lower reference limit, defined as the minimum fold change (FC) value of 7.41. The fold change ratio for each value was calculated by comparing the specific value to this lower limit.

To assess the impact of CIPC expression on OS, we utilized a threshold of 1.29 to define overexpression. This value was determined based on the median CIPC expression level relative to the minimum FC value, with the interquartile range (IQR) added to this baseline. The selection of 1.29 as the cutoff point aimed to identify cases with significantly elevated CIPC expression, allowing for a more precise examination of its potential correlation with patient outcomes.

## 5. Results

### 5.1. Literature Review and Bioinformatic Analysis Approach

To better understand the role of *CIPC* in circadian rhythms (CRs) and to outline its possible involvement in leukemogenesis, a search was conducted in the PubMed database using the keywords “CIPC”, “CLOCK-interacting protein, Circadian (CIPC)”, and “CLOCK-interacting pacemaker, Circadian (CIPC)”. Initially, six articles were found. Given the small number of studies, additional searches were performed in other databases, such as Scientific Electronic Library Online (SciELO) and Google Scholar, yielding a total of 89 works. We applied strict exclusion criteria due to the high number of reviews, abstracts, or articles not published in peer-reviewed journals. Therefore, only previously published clinical or preclinical studies were included, where study models involved invertebrate animals, animals, or human cells. A total of eight studies met these criteria and are summarized in Table 1.

**Figure 1:**
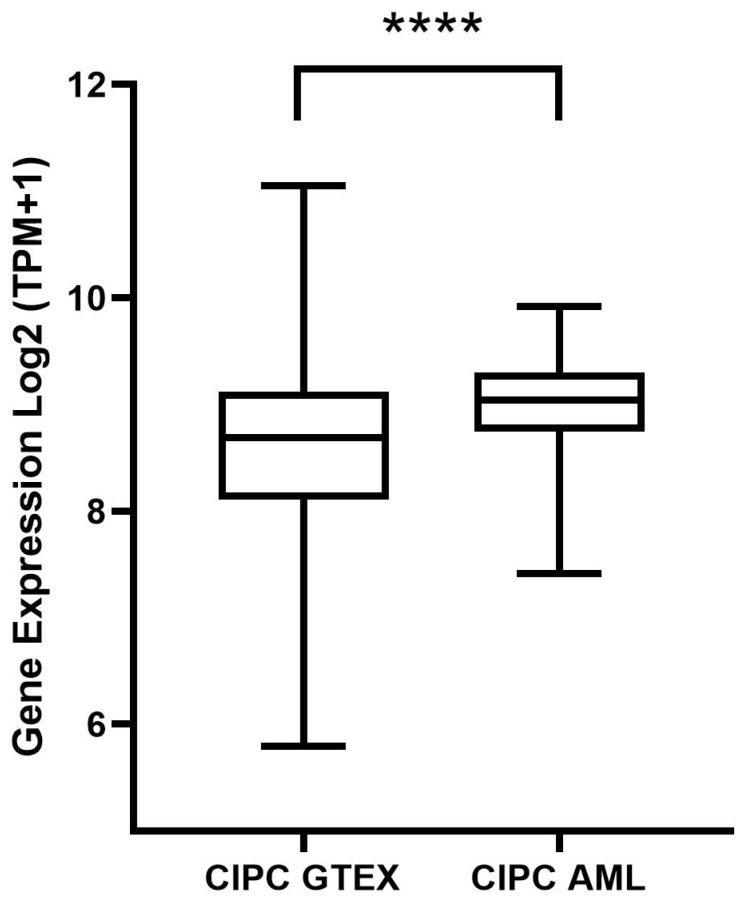
Gene expression analysis of CIPC in relation to healthy tissue. Legend: Gene expression analysis of *CIPC* using acute myeloid leukemia as the differential neoplastic tissue (*n* = 173) from Xena Browser (TCGA-LAML), with the normal tissue (*n* = 337) from the GTEx (Genotype-Tissue Expression Project), was performed using log_2_-transformed transcripts per million (TPM + 1). The *p*-value was *p* < 0.0001 ****. This analysis was conducted using GraphPad Prism version 8.0.1.

**Figure 2:**
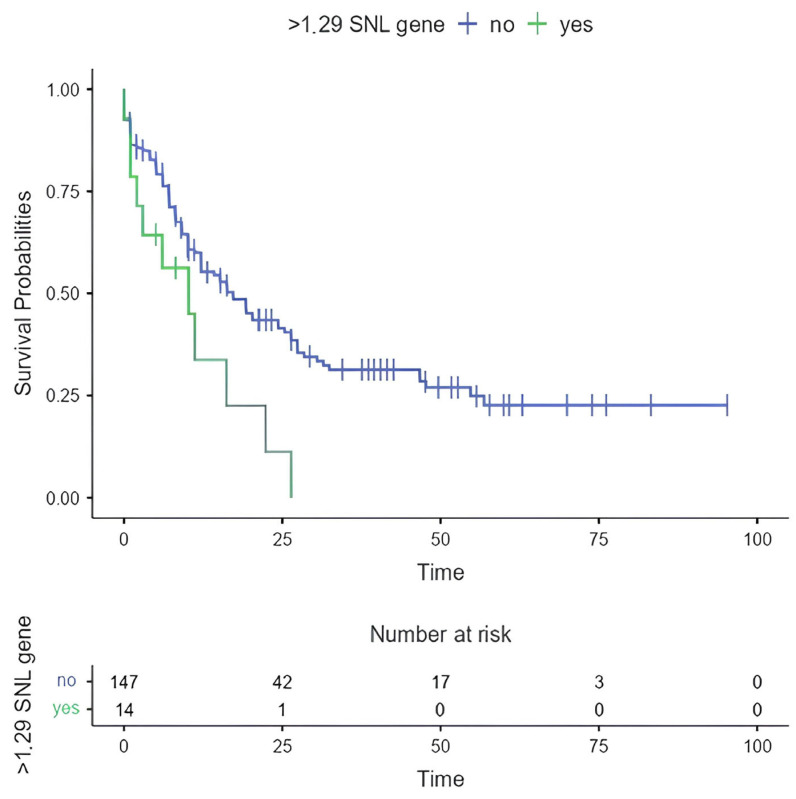
Overall survival based on overexpression of *CIPC* based on minimum fold change. Legend: The Kaplan–Meier survival curve illustrates the survival outcomes of patients with acute myeloid leukemia stratified by expression levels of the *CIPC* gene. Statistical analysis using the log-rank test yielded a *p*-value of 0.014, indicating a significant difference in survival between the groups with varying *CIPC* expression. Additionally, the hazard ratio (HR) was 2.15, with a corresponding *p*-value of 0.017, further supporting the presence of a statistically significant association between elevated *CIPC* expression and reduced survival in AML patients. These analyses were conducted using data obtained from the Xena platform.

The eight studies demonstrate *CIPC* activity in CRs. Given the well-established deregulation of CGs in cancer, we decided to analyze CIPC expression levels in human tumors using the Xena Browser [62], which imports data from The Cancer Genome Atlas (TCGA) and the Genotype-Tissue Expression (GTEx) project, thus providing a reliable database for comparing expression levels between tumors and healthy tissues. An image was generated containing expression comparisons across 34 tumor types available in the tool (Figure 1), along with a boxplot specifically depicting *CIPC* expression in AML (Figure 1). Furthermore, all analyses were performed using the Ensembl *CIPC* ID (ENSG00000198894) [63] for standardization. Additionally, the Xena Browser was used to evaluate the survival of patients with *CIPC*–expressing AML, generating a Kaplan–Meier survival curve comparing overexpression and low expression groups (Figure 2).

### 5.2. Gene Expression Analysis of CIPC and Survival Analysis

The results of the *CIPC* gene expression analysis (Figure 1) demonstrate a statistically significant difference (*p* < 0.0001 ****), indicating that *CIPC* is overexpressed in AML tissues compared to non-neoplastic tissues.

The survival analysis (Figure 2) revealed a statistically significant difference in OS between patients with varying levels of *CIPC* expression (log-rank test: *p* = 0.014; HR = 2.15; *p* = 0.017). These findings suggest a potential association between elevated CIPC expression and reduced survival rates. The Cox proportional hazards model further supports this association, indicating that higher *CIPC* expression correlates with a significantly increased risk of adverse clinical outcomes. Together, these results highlight the potential prognostic value of *CIPC* expression in AML.

However, although the observed associations are statistically significant, these findings should be interpreted with caution due to the limited number of studies involving leukemia patients. Further research is necessary to clarify the role of CIPC in survival outcomes and to validate these preliminary results.

Emerging evidence suggests that hyperactivity of the CIPC molecule may represent a promising therapeutic target. Targeting this hyperactivity could potentially serve as an effective strategy for both risk stratification and treatment. The impact of high expression was notable in our analysis, whereas CIPC hypoactivity did not show immediate detrimental effects (*p* = 0.3); however, its clinical significance remains to be fully understood.

Before considering any therapeutic interventions aimed at modulating CIPC activity, a more comprehensive understanding of its role in leukemia pathophysiology is required.

### 5.3. Functional Network Analysis of CIPC Using STRING and GENEMANIA

**Figure 3:**
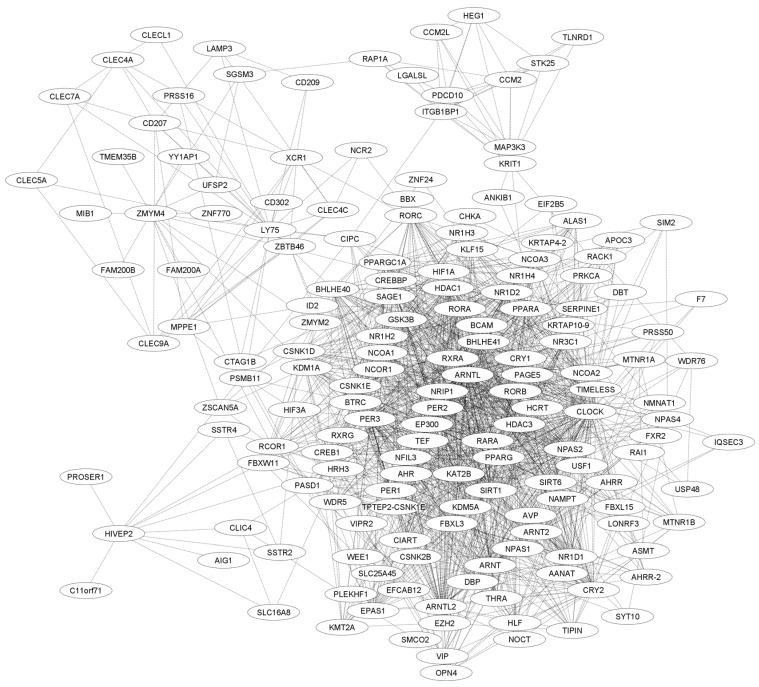
Interaction network generated by the STRING database with identification of functional clusters from *CIPC*. Legend: Interaction generated by the STRING database from 169 genes correlated with CIPC. These interactions are derived from databases and experimental data, as well as predicted interactions. A network analysis was performed from this network to identify the main clusters involved in the CIPC pathways.

**Figure 4:**
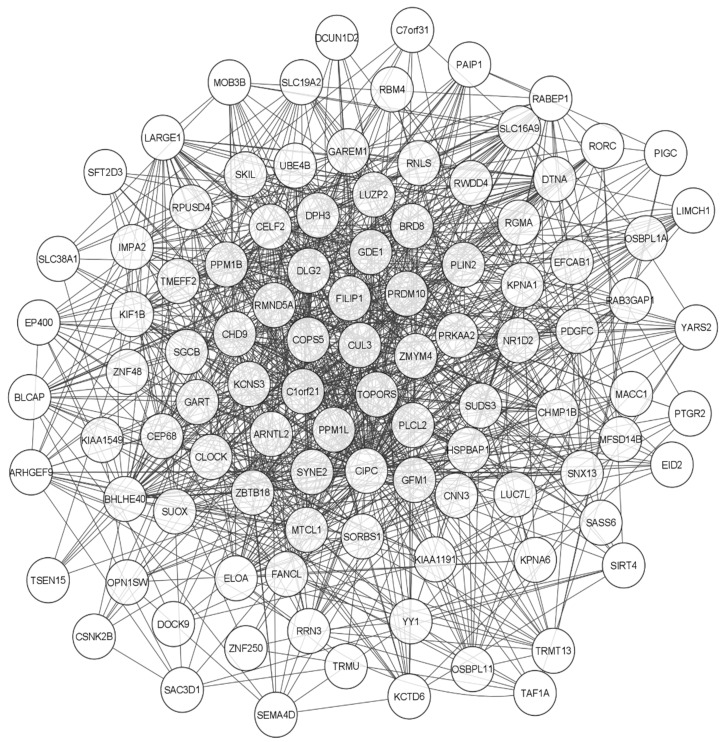
Interaction network generated by GENEMANIA with identification of functional clusters from CIPC. Legend: Interaction generated by the GENEMANIA database from 101 genes correlated with CIPC. These interactions are based on coexpression data, physical interactions, genetic alterations, co-localization, shared protein domains, genomic neighborhood, and gene ontology annotation. A network analysis was performed on this network to identify the main clusters involved in the CIPC pathway.

In this study, different bioinformatics tools were also used to evaluate the potential functions of CIPC in other pathways that may influence human carcinogenesis, specifically leukemogenesis. The STRING database [64] was used to generate a network of protein–protein interactions, and the results were imported into CYTOSCAPE version 3.10.2 [65] for further analysis (Figure 3). Additionally, another interaction network genetic interaction was generated using the GENEMANIA database [66]. Using the plugin available in CYTOSCAPE version 3.10.2, these networks were constructed and employed to jointly evaluate pathway interactions, physical interactions, coexpression, genetic interactions, and target predictions (Figure 4) [65].

Using the data generated by the databases, a network analysis was performed using the tools available in CYTOSCAPE, resulting in two tables that were exported and analyzed in Microsoft Excel.

From the interaction analysis, the genes with the highest degree in both pathways were filtered and evaluated. Subsequently, the DAVID database [67,68] was used to perform functional annotation using KEGG, REACTOME, and WIKIPATHWAYS, aiming to identify which pathways CIPC may influence and to highlight those already known to be associated with cancer. As a result, eight proteins stood out in the analyses. This information was then used to predict potential mechanisms by which CIPC may contribute to leukemogenic development. The data are presented in Table 2.

## 6. Discussion

*CIPC* is a key regulator of the mammalian circadian clock system. This protein functions as a negative feedback regulator that specifically inhibits the activity of the CLOCK–BMAL1 complex, a core component of the circadian machinery. Notably, CIPC exerts its inhibitory effect independently of other known negative regulators of the circadian system, such as CRYs [52,53,54].

*CIPC* is expressed in several tissues and forms complexes with *CLOCK* in vivo. When endogenous *CIPC* is depleted, the circadian period becomes shortened, indicating its essential role in maintaining an adequate circadian rhythm duration. Furthermore, unlike other circadian genes, *CIPC* has no known homologues in invertebrates, being unique to vertebrates and suggesting that it represents an evolutionary addition to the complexity of the vertebrate circadian clock system [52,53,54].

This negative regulator may stimulate BMAL1–dependent phosphorylation of CLOCK. A mutant CLOCK protein lacking the CIPC–binding domain exhibited a reduced capacity for phosphorylation and subsequent degradation when compared to the wild-type protein. These findings suggest that negative feedback-mediated CLOCK phosphorylation contributes to both E-box-dependent transcriptional activation and CLOCK degradation [59].

In this context, it is worth noting that the CLOCK–BMAL1 complex is negatively regulated across all types of leukemia [50]. Our analyses showed that CIPC may be overexpressed in AML, suggesting that this negative regulation could be influenced by CIPC. Furthermore, not only was CLOCK observed to have altered regulation in leukemias, but its analogue, the NPAS2 gene, was also found to be overexpressed in AML [69].

Although the expression of rs2305160 was not significant, other studies have shown that polymorphisms in this gene may be associated with risk biomarkers, such as in lung cancer and potentially in lymphomas and breast cancer [70,71].

Incidentally, Yoshitane and Fukada [60] demonstrated that CIPC can influence NPAS2, likely through the same mechanisms by which it negatively regulates CLOCK. Furthermore, another study by Yoshitane [59] suggested that *CIPC* may also regulate BMAL1, indicating a central negative regulatory role beyond its direct interaction with CLOCK.

The negative regulation of *CIPC* on the CLOCK–BMAL1 and NPAS2–BMAL1 complexes is now well established. However, studies have demonstrated that CIPC is not critically required for CRs, suggesting that CIPC may have versatile functions independent of circadian regulation [52,54,60].

Therefore, Matsunaga et al. observed in studies using cell cultures and mouse models that overexpression of CIPC reduces the activation of Extracellular Signal-Regulated Kinase (ERK), while the absence of CIPC results in increased ERK activity. This demonstrates a versatile function of CIPC, integrating circadian rhythms with cell signaling [52]. In this context, it is well established that aberrant regulation of the Raf/MEK/ERK pathways can contribute to uncontrolled cell growth in leukemia [72,73] thus suggest another possible oncogenic role for this circadian negative regulator.

It is noteworthy that Janssen [74] observed ERK pathway activity as an important factor in the synergistic effect of the combination of Venetoclax and Gilteritinib in patients with *FLT3* wild-type high-risk AML, and how these drugs work together to suppress the MCL-1 protein. Along with *ERK,* the study also observed a correlation with *GSK3B*, an important protein with complex functions that can influence cell proliferation, survival, and apoptosis. In our bioinformatics analyses, we found significant interactions between *CIPC* and *GSK3B*, which may support this possible versatile function of *CIPC* [74,75].

Another interesting finding in the interaction networks was the high degree of *EZH2* in the network generated from interactions with *CIPC*. This gene has already been described and may act as either an oncogene or tumor suppressor in hematological malignancies. *EZH2* loss-of-function mutations are common in patients with myelodysplastic/myeloproliferative neoplasms, myelodysplastic syndrome, and myelofibrosis. In cases of chronic myeloid leukemia (CML), *EZH2* is often characterized by its overexpression [76,77].

Furthermore, studies show that *EZH2* is necessary for the function of the circadian clock in zebrafish, and that its deficiency results in significant disruption of the circadian rhythm. Along with its circadian role, *EZH2* is also crucial for the proliferation and differentiation of hematopoietic progenitor cells in zebrafish [78].

Another high-degree finding from our CIPC network analyses, as shown in Table 2, is the SKIL protein, also known as SnoN. SKIL is an oncogene belonging to the SKI family and is involved in regulating several cell signaling pathways, including the TGF-β pathway. Its overexpression has been associated with progression and treatment resistance in chronic myeloid leukemia (CML) [79,80]. *WDR5* is a key component protein of the Mixed Lineage Leukemia (MLL) histone methyltransferase complex, which is crucial for the regulation of gene transcription. It is highly expressed in various leukemias, acting as an oncogene that promotes the control of leukemic cells and contributes to tumorigenesis [81,82].

CSNK2B promotes cell regulation by activating the mTOR signaling pathway in cancers [83]. SIRT6 facilitates responses to chemotherapy in leukemia cells, helping them survive treatment-induced stress [84]; EP400 has been shown to be crucial in sustaining the oncogenic potential of MLL leukemia stem cells [85].

## 7. Conclusions

The expression levels and function of CIPC remain unclear. In this study, using bioinformatics approaches, we demonstrated a potential aberrant expression of this protein in leukemic patients, suggesting a possible leukemogenic role. CIPC clearly inhibits key genes of the circadian rhythm, which are consistently altered across all types of leukemia. Beyond this inhibition, our data revealed interactions between CIPC and other critical oncogenic pathways, such as the MAPK/ERK pathway, known for its role in cell proliferation and differentiation. Although these findings underscore the relevance of CIPC in AML, further studies are necessary to validate these results and elucidate the precise mechanistic role of CIPC in cancer. This work contributes to a deeper understanding of how circadian regulation impacts cancer progression and offers new insights for potential therapeutic strategies.

## Figures and Tables

**Table 1:** Overview of studies investigating the role of CIPC in the circadian cycle and cancer.

STUDY MODEL	FUNCTION	REFERENCE
DROSOPHILA	CIPC represents an alternative and specific mechanism of transcriptional repression within the molecular clock, distinct from the CRY– and PER–mediated pathways. Its unique structural interaction with the CLOCK protein at exon 19 plays a crucial role in the complexity and precision of circadian regulation in mammals and some invertebrates.	[57]
DROSOPHILA	*CIPC* functions as a negative regulator of the *CLOCK–CYCLE (CLK-CYC)* complex, and its expression is suppressed by *CLOCKWORK ORANGE (CWO)* to facilitate effective circadian transcriptional activation. This modulation of *CIPC* expression by *CWO* represents an additional and crucial mechanism of transcriptional control within the circadian clock.	[58]
MICE	*CIPC* binds to *CLOCK* at an important site, inhibiting the transcriptional activity of the *CLOCK–BMAL1* heterodimer in mammalian cells.	[54]
MICE AND NIH 3T3 CELLS (mice fibroblast cell line)	*CIPC* stimulates *CLOCK* phosphorylation and increases *CLOCK* and *BMAL1* levels. Stabilization of *BMAL1* is not observed in the absence of coexpressed *CLOCK*. Coexpression of *CIPC* with *CLOCK* without BMAL1 expression had a marginal effect on phosphorylation levels. In *CLOCKΔ19,* a *CLOCK* mutant without the *CIPC*–binding region, *CIPC* influenced the stability of *BMAL1* in the CLOCKΔ19–BMAL1 complex without efficiently binding to CLOCKΔ19.	[59]
MICE AND NIH 3T3 CELLS	*CIPC* stimulates the phosphorylation of *CLOCK* in the CLOCK–BMAL1 complex as well as NPAS2 in the NPAS2–BMAL1 complex, probably through the same mechanisms.	[60]
MICE -/- (knockout) and WILD-TYPE MICE	*CIPC* does not function in determining the period in locomotor rhythms. It was observed that only the *PER1* peak in *CIPC-/-* mice was reduced to half the level compared to wild-type mice.	[53]
HEK293 CELLS (human kidney cell line)	Identification of amino acid residues Lys186 and Lys187 as essential for CIPC nuclear signaling. Identification of CIPC–binding proteins such as the enzyme carbamoyl-phosphate synthetase 2, aspartate transcarbamoylase, and dihydroorotase (CAD). Erk activation caused by phorbol 12-myristate 13-acetate (PMA) was inhibited with CIPC expression. CIPC subcellular localization was dramatically altered in cells synchronized at the G1/S boundary using a double thymidine blockade, suggesting translocation to the cytosol.	[52]
MDX MICE	*CIPC* is upregulated during myoblast differentiation. *CIPC* deficiency leads to activation of the *ERK1/2* and *JNK1/2* signaling pathways, which activates the transcription factor *SP1* and triggers the transcription of *Paired* Box 7 *(PAX7)* and *Myogenic Differentiation 1 (MYOD*)	[61]

Legend: The table summarizes the experimental studies that explore the potential roles of CIPC (CLOCK-interacting pacemaker) in circadian rhythms (CRs) and other pathways relevant to carcinogenesis. The selection criteria included all experimental work with human CR relevance or homologous interactions, excluding literature reviews and non-human specific studies.

**Table 2:** Functional annotation and pathway analysis of CIPC.

ID	GENE	PATHWAYS
ENSG00000183495	E1A binding protein p400 (*EP400*)	Cellular responses to stress, cellular senescence, DNA damage/telomere stress-induced senescence
ENSG00000136603	SKI like proto-oncogene (*SKIL*)	TGF–beta signaling pathway, transcriptional activity of SMAD2/SMAD3 heterotrimer
ENSG00000196363	WD repeat domain 5 (*WDR5*)	Epigenetic regulation of gene expression, chromatin-modifying enzymes, pleural mesothelioma
ENSG00000204435	Casein kinase 2 beta (*CSNK2B*)	NF-kappa B signaling pathway, PD-L1 expression and PD-1 checkpoint pathway in cancer, breast cancer pathway, lncRNA in canonical Wnt signaling and colorectal cancer, ncRNAs involved in Wnt signaling in hepatocellular carcinoma, pleural mesothelioma
ENSG00000106462	Enhancer of zeste 2 polycomb repressive complex 2 subunit (*EZH2*)	Polycomb repressive complex, microRNAs in cancer, cellular senescence, lncRNA in canonical Wnt signaling and colorectal cancer, ncRNAs involved in Wnt signaling in hepatocellular carcinoma, pleural mesothelioma
ENSG00000082701	Glycogen synthase kinase 3 beta (*GSK3B*)	Pathways in cancer, colorectal cancer, endometrial cancer, prostate cancer, breast cancer, hepatocellular carcinoma, gastric cancer, PI3K/AKT signaling in cancer, lncRNA in canonical Wnt signaling and colorectal cancer, ncRNAs involved in Wnt signaling in hepatocellular carcinoma
ENSG00000171720	Histone deacetylase 3 (*HDAC3*)	Viral carcinogenesis, signaling by NOTCH1 in cancer, HDACs deacetylate histones, chromatin-modifying enzymes
ENSG00000077463	Sirtuin 6 *(SIRT6)*	Central carbon metabolism in cancer

Legend: The results of the interaction analysis of genes with the highest degree in both pathways. Functional annotations were created using the DAVID database with KEGG, REACTOME, and WIKIPATHWAYS to identify pathways potentially influenced by CIPC and to filter those relevant to cancer. The table highlights eight key proteins identified in the analysis and predicts the possible mechanisms of CIPC involvement in leukemogenic development.

## References

[1] Huang R.C. (2018). The Discoveries of Molecular Mechanisms for the Circadian Rhythm: The 2017 Nobel Prize in Physiology or Medicine. Biomed. J..

[2] Jagannath A., Taylor L., Wakaf Z., Vasudevan S.R., Foster R.G. (2017). The Genetics of Circadian Rhythms, Sleep and Health. Hum. Mol. Genet..

[3] Konopka R.J., Benzer S. (1971). Clock Mutants of Drosophila Melanogaster (Eclosion/Circadian/Rhythms/X Chromosome). Proc. Natl. Acad. Sci. USA.

[4] Duan J., Greenberg E.N., Karri S.S., Andersen B. (2021). The Circadian Clock and Diseases of the Skin. FEBS Lett..

[5] Rosbash M. (2021). Circadian Rhythms and the Transcriptional Feedback Loop (Nobel Lecture)**. Angew. Chem. Int. Ed..

[6] Yang Y., Lindsey-Boltz L.A., Vaughn C.M., Selby C.P., Cao X., Liu Z., Hsu D.S., Sancar A. (2021). Circadian Clock, Carcinogenesis, Chronochemotherapy Connections. J. Biol. Chem..

[7] King D.P., Zhao Y., Sangoram A.M., Wilsbacher L.D., Tanaka M., Antoch M.P., Steeves T.D.L., Vitaterna M.H., Kornhauser J.M., Lowrey P.L. (1997). Positional Cloning of the Mouse Circadian Clock Gene. Cell.

[8] Kraft M., Martin R. (1995). Chronobiology and Chronotherapy in Medicine. Disease-a-Month.

[9] Reddy P., Zehring W.A., Wheeler D.A., Pirrotta V., Hadfield C., Hall J.C., Rosbash M. (1984). Molecular Analysis of the Period Locus in Drosophila Melanogaster and Identification of a Transcript Involved in Biological Rhythms. Cell.

[10] Sweeney B.M. (1983). Biological Clocks-An Introduction. BioScience.

[11] Hastings M.H., Maywood E.S., Brancaccio M. (2018). Generation of Circadian Rhythms in the Suprachiasmatic Nucleus. Nat. Rev. Neurosci..

[12] Ruan W., Yuan X., Eltzschig H.K. (2021). Circadian Rhythm as a Therapeutic Target. Nat. Rev. Drug Discov..

[13] Wang X.L., Li L. (2021). Circadian Clock Regulates Inflammation and the Development of Neurodegeneration. Front. Cell. Infect. Microbiol..

[14] Cao X., Yang Y., Selby C.P., Liu Z., Sancar A. (2021). Molecular Mechanism of the Repressive Phase of the Mammalian Circadian Clock. Proc. Natl. Acad. Sci. USA.

[15] Sinturel F., Gos P., Petrenko V., Hagedorn C., Kreppel F., Storch K.F., Knutti D., Liani A., Weitz C., Emmenegger Y. (2021). Circadian Hepatocyte Clocks Keep Synchrony in the Absence of a Master Pacemaker in the Suprachiasmatic Nucleus or Other Extrahepatic Clocks. Genes Dev..

[16] Ray S., Valekunja U.K., Stangherlin A., Howell S.A., Snijders A.P., Damodaran G., Reddy A.B. (2020). Circadian Rhythms in the Absence of the Clock Gene Bmal1. Science.

[17] Takahashi J.S. (2016). Transcriptional Architecture of the Mammalian Circadian Clock. Nat. Rev. Genet..

[18] Allada R., Bass J. (2021). Circadian Mechanisms in Medicine. N. Engl. J. Med..

[19] Chen H., Gao L., Yang D., Xiao Y., Zhang M., Li C., Wang A., Jin Y. (2019). Coordination between the Circadian Clock and Androgen Signaling Is Required to Sustain Rhythmic Expression of Elovl3 in Mouse Liver. J. Biol. Chem..

[20] Rutter J., Reick M., Wu L.C., Mcknight S.L. (2001). Regulation of Clock and NPAS2 DNA Binding by the Redox State of NAD Cofactors. Science.

[21] Poggiogalle E., Jamshed H., Peterson C.M. (2018). Circadian Regulation of Glucose, Lipid, and Energy Metabolism in Humans. Metabolism.

[22] Li Q., Zhang S., Wang H., Wang Z., Zhang X., Wang Y., Yuan J. (2023). Association of Rotating Night Shift Work, CLOCK, MTNR1A, MTNR1B Genes Polymorphisms and Their Interactions with Type 2 Diabetes among Steelworkers: A Case–Control Study. BMC Genom..

[23] Marcheva B., Ramsey K.M., Buhr E.D., Kobayashi Y., Su H., Ko C.H., Ivanova G., Omura C., Mo S., Vitaterna M.H. (2010). Disruption of the Clock Components CLOCK and BMAL1 Leads to Hypoinsulinaemia and Diabetes. Nature.

[24] Cai Y., Liu S., Sothern R.B., Xu S., Chan P. (2010). Expression of Clock Genes Per1 and Bmal1 in Total Leukocytes in Health and Parkinson’s Disease. Eur. J. Neurol..

[25] Niu L., Zhang F., Xu X., Yang Y., Li S., Liu H., Le W. (2022). Chronic Sleep Deprivation Altered the Expression of Circadian Clock Genes and Aggravated Alzheimer’s Disease Neuropathology. Brain Pathol..

[26] Farshadi E., van der Horst G.T.J., Chaves I. (2020). Molecular Links between the Circadian Clock and the Cell Cycle. J. Mol. Biol..

[27] Rijo-Ferreira F., Takahashi J.S. (2019). Genomics of Circadian Rhythms in Health and Disease. Genome Med..

[28] Gauger M.A., Sancar A. (2005). Cryptochrome, Circadian Cycle, Cell Cycle Checkpoints, and Cancer. Cancer Res..

[29] Samoilova E.M., Belopasov V.V., Ekusheva E.V., Zhang C., Troitskiy A.V., Baklaushev V.P. (2021). Epigenetic Clock and Circadian Rhythms in Stem Cell Aging and Rejuvenation. J. Pers. Med..

[30] Uchida Y., Hirayama J., Nishina H. (2010). A Common Origin: Signaling Similarities in the Regulation of the Circadian Clock and DNA Damage Responses Current Topics Review. Biol. Pharm. Bull..

[31] Shostak A. (2017). Circadian Clock, Cell Division, and Cancer: From Molecules to Organism. Int. J. Mol. Sci..

[32] Hanahan D. (2022). Hallmarks of Cancer: New Dimensions. Cancer Discov..

[33] Hanahan D., Weinberg R.A. (2000). The Hallmarks of Cancer Review Evolve Progressively from Normalcy via a Series of Pre. Cell.

[34] Hanahan D., Weinberg R.A. (2011). Hallmarks of Cancer: The next Generation. Cell.

[35] Gyamfi J., Kim J., Choi J. (2022). Cancer as a Metabolic Disorder. Int. J. Mol. Sci..

[36] Papagiannakopoulos T., Bauer M.R., Davidson S.M., Heimann M., Subbaraj L., Bhutkar A., Bartlebaugh J., Vander Heiden M.G., Jacks T. (2016). Circadian Rhythm Disruption Promotes Lung Tumorigenesis. Cell Metab..

[37] Altman B.J., Hsieh A.L., Sengupta A., Krishnanaiah S.Y., Stine Z.E., Walton Z.E., Gouw A.M., Venkataraman A., Li B., Goraksha-Hicks P. (2015). MYC Disrupts the Circadian Clock and Metabolism in Cancer Cells. Cell Metab..

[38] Schernhammer E.S., Razavi P., Li T.Y., Qureshi A.A., Han J. (2011). Rotating Night Shifts and Risk of Skin Cancer in the Nurses’ Health Study. J. Natl. Cancer Inst..

[39] Hu M.L., Yeh K.T., Lin P.M., Hsu C.M., Hsiao H.H., Liu Y.C., Lin H.Y.H., Lin S.F., Yang M.Y. (2014). Deregulated Expression of Circadian Clock Genes in Gastric Cancer. BMC Gastroenterol..

[40] Karantanos T., Theodoropoulos G., Pektasides D., Gazouli M. (2014). Clock Genes: Their Role in Colorectal Cancer. World J. Gastroenterol..

[41] Gréchez-Cassiau A., Rayet B., Guillaumond F., Teboul M., Delaunay F. (2008). The Circadian Clock Component BMAL1 Is a Critical Regulator of P21 WAF1/CIP1 Expression and Hepatocyte Proliferation. J. Biol. Chem..

[42] Qu M., Zhang G., Qu H., Vu A., Wu R., Tsukamoto H., Jia Z., Huang W., Lenz H.J., Rich J.N. (2023). Circadian Regulator BMAL1::CLOCK Promotes Cell Proliferation in Hepatocellular Carcinoma by Controlling Apoptosis and Cell Cycle. Proc. Natl. Acad. Sci. USA.

[43] Juliusson G., Hough R. (2016). Leukemia. Prog. Tumor Res..

[44] Du M., Chen W., Liu K., Wang L., Hu Y., Mao Y., Sun X., Luo Y., Shi J., Shao K. (2022). The Global Burden of Leukemia and Its Attributable Factors in 204 Countries and Territories: Findings from the Global Burden of Disease 2019 Study and Projections to 2030. J. Oncol..

[45] Huang F., Guang P., Li F., Liu X., Zhang W., Huang W., Haque N. (2020). AML, ALL, and CML Classification and Diagnosis Based on Bone Marrow Cell Morphology Combined with Convolutional Neural Network: A STARD Compliant Diagnosis Research. Medicine.

[46] Salama M.M., Aborehab N.M., El Mahdy N.M., Zayed A., Ezzat S.M. (2023). Nanotechnology in Leukemia: Diagnosis, Efficient-Targeted Drug Delivery, and Clinical Trials. Eur. J. Med. Res..

[47] Iqbal Z. (2012). Molecular Hematology in Leukemia Biology and Treatment: Past, Present, and Future. J. Appl. Hematol..

[48] Li H.X. (2019). The Role of Circadian Clock Genes in Tumors. Onco Targets Ther..

[49] Rasheed A. (2022). Niche Regulation of Hematopoiesis: The Environment Is “Micro,” but the Influence Is Large. Arterioscler. Thromb. Vasc. Biol..

[50] Sanford A.B.A., da Cunha L.S., Machado C.B., de Pinho Pessoa F.M.C., Silva A.N.d.S., Ribeiro R.M., Moreira F.C., de Moraes Filho M.O., de Moraes M.E.A., de Souza L.E.B. (2022). Circadian Rhythm Dysregulation and Leukemia Development: The Role of Clock Genes as Promising Biomarkers. Int. J. Mol. Sci..

[51] Puram R.V., Kowalczyk M.S., De Boer C.G., Schneider R.K., Miller P.G., McConkey M., Tothova Z., Tejero H., Heckl D., Järås M. (2016). Core Circadian Clock Genes Regulate Leukemia Stem Cells in AML. Cell.

[52] Matsunaga R., Nishino T., Yokoyama A., Nakashima A., Kikkawa U., Konishi H. (2016). Versatile Function of the Circadian Protein CIPC as a Regulator of Erk Activation. Biochem. Biophys. Res. Commun..

[53] Qu Z.P., Wang X.H., Liu D.C., Gao X., Xu Y. (2015). Inactivation of Cipc Alters the Expression of Per1 but Not Circadian Rhythms in Mice. Sci. China Life Sci..

[54] Zhao W.N., Malinin N., Yang F.C., Staknis D., Gekakis N., Maier B., Reischl S., Kramer A., Weitz C.J. (2007). CIPC Is a Mammalian Circadian Clock Protein without Invertebrate Homologues. Nat. Cell Biol..

[55] Griffin E.A., Staknis D., Weitz C.J. (1999). Weitz Light-Independent Role of CRY1 and CRY2 in the Mammalian Circadian Clock. Science.

[56] Li M.D., Xin H., Yuan Y., Yang X., Li H., Tian D., Zhang H., Zhang Z., Han T.L., Chen Q. (2021). Circadian Clock-Controlled Checkpoints in the Pathogenesis of Complex Disease. Front. Genet..

[57] Hou Z., Su L., Pei J., Grishin N.V., Zhang H. (2017). Crystal Structure of the CLOCK Transactivation Domain Exon19 in Complex with a Repressor. Structure.

[58] Rivas G.B.S., Zhou J., Merlin C., Hardin P.E. (2021). CLOCKWORK ORANGE Promotes CLOCK-CYCLE Activation via the Putative Drosophila Ortholog of CLOCK INTERACTING PROTEIN CIRCADIAN. Curr. Biol..

[59] Yoshitane H., Takao T., Satomi Y., Du N.-H., Okano T., Fukada Y. (2009). Roles of CLOCK Phosphorylation in Suppression of E-Box-Dependent Transcription. Mol. Cell. Biol..

[60] Yoshitane H., Fukada Y. (2009). CIPC-Dependent Phosphorylation of CLOCK and NPAS2 in Circadian Clockwork. Sleep Biol. Rhythm..

[61] Zheng J., Lou J., Li Y., Qian P., He W., Hao Y., Xue T., Li Y., Song Y.H. (2022). Satellite Cell-Specific Deletion of Cipc Alleviates Myopathy in Mdx Mice. Cell Rep..

[62] Goldman M.J., Craft B., Hastie M., Repečka K., McDade F., Kamath A., Banerjee A., Luo Y., Rogers D., Brooks A.N. (2020). Visualizing and Interpreting Cancer Genomics Data via the Xena Platform. Nat. Biotechnol..

[63] Harrison P.W., Amode M.R., Austine-Orimoloye O., Azov A.G., Barba M., Barnes I., Becker A., Bennett R., Berry A., Bhai J. (2024). Ensembl 2024. Nucleic Acids Res..

[64] Szklarczyk D., Franceschini A., Wyder S., Forslund K., Heller D., Huerta-Cepas J., Simonovic M., Roth A., Santos A., Tsafou K.P. (2015). STRING V10: Protein-Protein Interaction Networks, Integrated over the Tree of Life. Nucleic Acids Res..

[65] Shannon P., Markiel A., Ozier O., Baliga N.S., Wang J.T., Ramage D., Amin N., Schwikowski B., Ideker T. (2003). Cytoscape: A Software Environment for Integrated Models of Biomolecular Interaction Networks. Genome Res..

[66] Mostafavi S., Ray D., Warde-Farley D., Grouios C., Morris Q. (2008). GeneMANIA: A Real-Time Multiple Association Network Integration Algorithm for Predicting Gene Function. Genome Biol..

[67] Huang D.W., Sherman B.T., Lempicki R.A. (2009). Systematic and Integrative Analysis of Large Gene Lists Using DAVID Bioinformatics Resources. Nat. Protoc..

[68] Sherman B.T., Hao M., Qiu J., Jiao X., Baseler M.W., Lane H.C., Imamichi T., Chang W. (2022). DAVID: A Web Server for Functional Enrichment Analysis and Functional Annotation of Gene Lists (2021 Update). Nucleic Acids Res..

[69] Song B., Chen Y., Liu Y., Wan C., Zhang L., Zhang W. (2019). NPAS2 Regulates Proliferation of Acute Myeloid Leukemia Cells via CDC25A-Mediated Cell Cycle Progression and Apoptosis. J. Cell. Biochem..

[70] Zhu Y., Stevens R.G., Leaderer D., Hoffman A., Holford T., Zhang Y., Brown H.N., Zheng T. (2007). Non-Synonymous Polymorphisms in the Circadian Gene NPAS2 and Breast Cancer Risk. Breast Cancer Res. Treat..

[71] Zhu Y., Leaderer D., Guss C., Brown H.N., Zhang Y., Boyle P., Stevens R.G., Hoffman A., Qin Q., Han X. (2007). Ala394Thr Polymorphism in the Clock Gene NPAS2: A Circadian Modifier for the Risk of Non-Hodgkin’s Lymphoma NIH Public Access. Int. J. Cancer.

[72] Steelman L.S., Abrams S.L., Whelan J., Bertrand F.E., Ludwig D.E., Bäsecke J., Libra M., Stivala F., Milella M., Tafuri A. (2008). Contributions of the Raf/MEK/ERK, PI3K/PTEN/Akt/MTOR and Jak/STAT Pathways to Leukemia. Leukemia.

[73] Zheng Z., Chen X., Zhang Y., Ren F., Ma Y. (2023). MEK/ERK and PI3K/AKT Pathway Inhibitors Affect the Transformation of Myelodysplastic Syndrome into Acute Myeloid Leukemia via H3K27me3 Methylases and Demethylases. Int. J. Oncol..

[74] Janssen M., Schmidt C., Bruch P.-M., Blank M.F., Rohde C., Waclawiczek A., Heid D., Renders S., Göllner S., Vierbaum L. (2022). Venetoclax Synergizes with Gilteritinib in FLT3 Wild-Type High-Risk Acute Myeloid Leukemia by Suppressing MCL-1. Blood.

[75] Wu H., Lu X.X., Wang J.R., Yang T.Y., Li X.M., He X.S., Li Y., Ye W.L., Wu Y., Gan W.J. (2019). TRAF6 Inhibits Colorectal Cancer Metastasis through Regulating Selective Autophagic CTNNB1/β-Catenin Degradation and Is Targeted for GSK3B/GSK3β-Mediated Phosphorylation and Degradation. Autophagy.

[76] Rinke J., Chase A., Cross N.C.P., Hochhaus A., Ernst T. (2020). EZH2 in Myeloid Malignancies. Cells.

[77] Safaei S., Baradaran B., Hagh M.F., Alivand M.R., Talebi M., Gharibi T., Solali S. (2018). Double Sword Role of EZH2 in Leukemia. Biomed. Pharmacother..

[78] Zhong Y., Ye Q., Chen C., Wang M., Wang H. (2018). Ezh2 Promotes Clock Function and Hematopoiesis Independent of Histone Methyltransferase Activity in Zebrafish. Nucleic Acids Res..

[79] Abdulmawjood B., Costa B., Roma-rodrigues C., Baptista P.V., Fernandes A.R. (2021). Genetic Biomarkers in Chronic Myeloid Leukemia: What Have We Learned so Far?. Int. J. Mol. Sci..

[80] Chi C., Liang X., Cui T., Gao X., Liu R., Yin C. (2023). SKIL/SnoN Attenuates TGF-Β1/SMAD Signaling-Dependent Collagen Synthesis in Hepatic Fibrosis. Biomol. Biomed..

[81] Ye X., Chen G., Jin J., Zhang B., Wang Y., Cai Z., Ye F. (2020). The Development of Inhibitors Targeting the Mixed Lineage Leukemia 1 (MLL1)-WD Repeat Domain 5 Protein (WDR5) Protein- Protein Interaction. Curr. Med. Chem..

[82] Ge Z., Song E.J., Kawasawa Y.I., Li J., Dovat S., Song C. (2016). WDR5 High Expression and Its Effect on Tumorigenesis in Leukemia. Oncotarget.

[83] Yu S., Hu Q., Fan K., Yang C., Gao Y. (2021). CSNK2B Contributes to Colorectal Cancer Cell Proliferation by Activating the MTOR Signaling. J. Cell Commun. Signal..

[84] Kong Q., Li Y., Liang Q., Xie J., Li X., Fang J. (2020). SIRT6-PARP1 Is Involved in HMGB1 PolyADP-Ribosylation and Acetylation and Promotes Chemotherapy-Induced Autophagy in Leukemia. Cancer Biol. Ther..

[85] Huang X., Spencer G.J., Lynch J.T., Ciceri F., Somerville T.D.D., Somervaille T.C.P. (2013). Enhancers of Polycomb EPC1 and EPC2 Sustain the Oncogenic Potential of MLL Leukemia Stem Cells. Leukemia.

